# Recognition and control gaps in advanced cardiovascular-kidney-metabolic syndrome among United States adults

**DOI:** 10.1016/j.pmedr.2026.103635

**Published:** 2026-09-20

**Authors:** Menghao Zhou, Fen Zhang, Bingluo Zhou, Yiming Lv

**Affiliations:** a Health Management Center, The First Affiliated Hospital, Zhejiang University School of Medicine, Hangzhou, Zhejiang, China; b Department of Geriatrics, The First Affiliated Hospital, Zhejiang University School of Medicine, Hangzhou, Zhejiang, China; c Department of Radiation Oncology, The Second Affiliated Hospital, Zhejiang University School of Medicine, Hangzhou, Zhejiang, China; d Department of Colorectal Surgery, Sir Run Run Shaw Hospital, Zhejiang University School of Medicine, Hangzhou, Zhejiang, China

**Keywords:** Cardiovascular-kidney-metabolic syndrome, Chronic kidney disease, Disease recognition, Risk factor control, Surveillance

## Abstract

**Objective:**

To describe cardiovascular-kidney-metabolic (CKM) stages and condition-specific indicators among adults in the United States, with exploratory state profiles.

**Methods:**

We analyzed 5006 United States adults aged ≥20 years in the National Health and Nutrition Examination Survey, August 2021–August 2023. Survey weights and condition-specific denominators were used. A stage-3 model developed among adults without reported cardiovascular disease was applied to the 2023 Behavioral Risk Factor Surveillance System.

**Results:**

Advanced CKM (stages 3–4) affected 13.0% (95% confidence interval, 11.6%, 14.4%) of adults. Among advanced-CKM adults with a single-visit kidney abnormality, 80.7% did not report weak or failing kidneys. Within applicable subgroups, 58.7% had blood pressure ≥ 130/80 mm Hg and 56.0% had glycated hemoglobin ≥7%. Concurrent elevation affected 34.1% (95% confidence interval, 24.7%, 44.4%) of the fixed hypertension-plus-diabetes subgroup (91/277). Across 49 jurisdictions, the age-standardized modeled stage-3 contribution ranged from 4.4% to 6.5%; directly reported cardiovascular disease was presented separately.

**Conclusions:**

National estimates describe frequent condition-specific abnormalities within advanced CKM, while the separate geographic projections provide exploratory surveillance context. Fixed thresholds do not establish individual treatment failure, and state projections require external validation.

## Highlights

• Advanced cardiovascular-kidney-metabolic syndrome affected 13.0% of adults.
• Kidney abnormalities were often not reported as weak or failing kidneys.
• Blood pressure and glycemic elevations were common in applicable subgroups.
• Joint blood pressure and glycemic elevation affected 34.1% of the fixed subgroup.
• State profiles separate reported disease from an exploratory modeled component.

## 1 Introduction

Cardiovascular-kidney-metabolic (CKM) syndrome links metabolic risk, chronic kidney disease (CKD), and cardiovascular disease (CVD) across a staged prevention continuum (Ndumele et al., 2023). A 2026 multisociety guideline further operationalized prevention, detection, evaluation, and management across CKM stages (Ndumele et al., 2026).

National studies have established CKM stage prevalence and its social gradients in United States adults (Aggarwal et al., 2024; Li et al., 2024; Minhas et al., 2024; Zhu et al., 2024). Risk-factor control in CKM-related populations is also well documented. Ostrominski et al. (2025) examined comprehensive control among adults with at least two of CVD, CKD, and type 2 diabetes. Lee et al. (2025) reported joint treatment and control among adults with hypertension and concurrent diabetes or hyperlipidemia through 2023. Gong et al. (2026a) assessed treatment and control in CKM stage ≥2 across cardiovascular-risk strata and cardiometabolic condition counts.

Kidney reporting, blood pressure, glycemia, and lipid-medication reporting require different clinical denominators. Assessing blood pressure and glycemic elevation in the same adults with hypertension and diabetes describes how often these abnormalities occur together. Access to care and food security provide social context for the clinical indicators.

The National Health and Nutrition Examination Survey (NHANES) provides measured national data, whereas the Behavioral Risk Factor Surveillance System (BRFSS), a telephone health survey, supports state estimates but lacks full staging measurements. Both represent the noninstitutionalized population of the United States. Cross-survey models can explore geographic patterns but require validation against measured state outcomes. Their 2021–2023 and 2023 data are contemporaneous (Terry et al., 2024). Previous state and neighborhood analyses provide context (Chang et al., 2025; Krishnan et al., 2026).

We used NHANES 2021–2023 to estimate CKM stages and characterize recognition, control, access, and food-security indicators within advanced CKM. BRFSS 2023 supported exploratory state profiles alongside cardiometabolic, social, and access descriptors. Joint indicators describe overlapping abnormalities; threshold, definition and selection sensitivities assess dependence on surveillance choices. Secondary comparisons assessed how differences in clinical indicators between stages 3 and 4 varied with demographic adjustment and restriction to groups represented in both stages.

## 2 Methods

### 2.1 Study design and data sources

We conducted a cross-sectional surveillance study with a secondary exploratory state application. Participant-level records were not pooled. NHANES August 2021–August 2023 provided measured national staging data and the development sample for a BRFSS-compatible bridge model (Terry et al., 2024). Among 5792 adults aged ≥20 years with positive phlebotomy weights and observed survey design variables, 786 lacked primary staging variables and were excluded, leaving 5006 complete cases (Supplementary Table S1). Four retained participants had unresolved risk-based staging and remained in the national distribution denominator. BRFSS 2023 supplied the state-representative application sample (Centers for Disease Control and Prevention, 2025). Kentucky and Pennsylvania were absent from the public-use file and excluded from state profiles.

NHANES interview and examination response rates were 34.6% and 25.7%, respectively (Terry et al., 2024). Across 48 states and the District of Columbia in BRFSS 2023, the median weighted American Association for Public Opinion Research Response Rate 4 was 44.6% (range, 31.0%–63.1%) (Centers for Disease Control and Prevention, 2024). Predictor harmonization appears in Supplementary Methods M4.

Survey-recorded sex (female or male) was used as available in the public-use datasets; gender identity was not included in the harmonized analyses.

### 2.2 CKM staging

We operationalized the American Heart Association CKM framework using measured body size, blood pressure, glycemia, lipid-related information, kidney markers, Predicting Risk of Cardiovascular Disease Events (PREVENT) risk, and CVD history (Khan et al., 2024; Ndumele et al., 2023). The highest captured stage was assigned. Mutually exclusive stages ranged from no captured risk factors (stage 0) to adiposity or prediabetes (stage 1), metabolic risk factors or a kidney abnormality (stage 2), very-high-risk kidney markers or PREVENT 10-year total CVD risk ≥20% without captured clinical CVD (stage 3), and reported clinical CVD (stage 4). PREVENT risk was used at ages 30–79 years without clinical CVD; outside this range, stage 3 required very-high-risk kidney markers. Advanced CKM comprised stages 3–4.

Estimated glomerular filtration rate (eGFR) was calculated using the 2021 race-free Chronic Kidney Disease Epidemiology Collaboration creatinine equation (Inker et al., 2021). Kidney abnormality meant single-visit eGFR <60 mL/min/1.73 m^2^ or urine albumin-to-creatinine ratio (UACR) ≥30 mg/g. The primary analysis used phlebotomy weights to avoid mixing fasting and nonfasting subsample weights. PREVENT used available optional glycated hemoglobin (HbA1c) and UACR inputs, without a social deprivation index. Required continuous risk inputs outside permitted ranges were bounded to the nearest American Heart Association-supported values for risk calculation alone; raw clinical measurements were preserved. This boundary convention was introduced during revision and can overestimate or underestimate risk (American Heart Association, 2026). Remaining uncomputable risks were not treated as low risk. Definitions and alternative specifications appear in Supplementary Methods M1 and Table S2.

### 2.3 Recognition, control, access, and social-risk indicators

Among adults with advanced CKM, we estimated: the proportion not reporting weak or failing kidneys (KIQ022) among those with a single-visit kidney abnormality; blood pressure ≥ 130/80 mm Hg (systolic ≥130 or diastolic ≥80) among adults with diagnosed or treated hypertension; HbA1c ≥7% among adults with diagnosed diabetes; and no reported lipid-lowering medication among the high-cholesterol proxy (total cholesterol ≥240 mg/dL or reported high cholesterol). Contextual indicators were no usual place for care, no health insurance, and low or very low food security among household adults (FSDAD). Each estimate required an observed indicator response; unknown kidney and food-security responses were excluded. Definitions and denominators appear in Supplementary Methods M1. Equivalent international-system units are provided in Supplementary Methods M1.

Concurrent elevation required both blood pressure ≥ 130/80 mm Hg and HbA1c ≥7% in the fixed subgroup with diagnosed/treated hypertension and diagnosed diabetes. Higher-threshold sensitivities used blood pressure ≥ 140/90 mm Hg (systolic ≥140 or diastolic ≥90) and HbA1c ≥8%, separately and jointly (Supplementary Fig. S1). Fixed thresholds support population surveillance (Fryar et al., 2024; Inoue et al., 2025); individual treatment targets vary with age, comorbidity, frailty, and treatment context.

### 2.4 Exploratory state-level bridge analysis and contextual profiles

The survey-weighted logistic bridge modeled operational stage 3 among NHANES adults without any captured clinical CVD, including heart failure. Predictors were age (natural spline, two degrees of freedom), an indicator for ages 30–79, sex, harmonized race/ethnicity, reported hypertension, diabetes, kidney disease, and current smoking. CVD was not a predictor. Training-fit spline bases were preserved for new predictions (Supplementary Methods M4).

Internal validation used 20 repetitions of five-fold primary sampling unit (PSU) cross-validation and 200 stratified PSU bootstrap optimism corrections. Discrimination, calibration, and reduced-model diagnostics appear in Supplementary Fig. S2 and Methods M4. Predictor-completeness inverse-probability weighting (IPW) used age, sex, race/ethnicity, and jurisdiction, with normalized predicted means within CVD-negative respondents (Supplementary Table S1).

BRFSS common CVD meant a report of myocardial infarction, coronary heart disease/angina, or stroke; any yes indicated CVD, all explicit no responses indicated no common CVD, and other patterns were unresolved. Within respondents with known common CVD, reported CVD prevalence and the modeled stage-3 contribution, (1 − reported CVD prevalence) × normalized mean prediction among common-CVD-negative respondents, were reported separately. Age standardization used the fixed pooled 49-jurisdiction BRFSS distribution. Intervals used delta-method variance propagation and a normal critical value of 1.96, combining BRFSS design covariance and NHANES coefficient variance. A finite-degree-of-freedom sensitivity assessed this approximation; both methods condition on response weights and the age reference (Supplementary Methods M5; Fig. S2).

Contextual indicators were obesity, diabetes, hypertension, reported kidney disease, poverty, insurance, rurality, primary-care physician density, food insecurity, and social vulnerability. Sources comprised federal public-health and census statistics, America's Health Rankings, and workforce and food-security reports (Flanagan et al., 2011; Greenlund et al., 2022; Health Resources and Services Administration, 2025; Rabbitt et al., 2024). Source units, periods and denominators remain distinct; no composite score or policy ranking is assigned (Supplementary Methods M6).

### 2.5 Equity analysis

Descriptive associations with advanced CKM were estimated using survey-weighted modified Poisson regression. A full model included age group, sex, race/ethnicity, education, family income relative to the federal poverty threshold, insurance, and food security. Comparison models used the same complete cases and included age (natural spline, two degrees of freedom), sex, race/ethnicity, and one social factor. They differ in both age specification and social-factor adjustment. Individual-coefficient intervals used design-degree-of-freedom t critical values consistently across both model sets. The full model provides supplementary description under more extensive adjustment; interpretation emphasizes patterns across specifications (Supplementary Table S3; Methods M3).

### 2.6 Statistical analysis

All analyses incorporated survey weights, strata, and PSUs. National proportions used Korn–Graubard beta 95% confidence intervals (CI), adjusted for each domain's design degrees of freedom; imprecise estimates carry reliability flags (Parker et al., 2017; Supplementary Methods M2). During revision, the NHANES analytic-selection model was expanded and refitted. IPW divided phlebotomy weights by completeness probabilities truncated at the complete-case 1st and 99th percentiles (Supplementary Table S1; Methods M2).

Analyses added during revision included alternative clinical thresholds, fixed-subgroup joint indicators, matched fasting definitions with fasting weights, PREVENT without optional HbA1c/UACR, and supporting adjusted comparisons. Fasting-defined diabetes was propagated into PREVENT. Secondary stage comparisons used age, sex, and race/ethnicity adjustment with explicitly defined standardization populations and each fitted model's residual design degrees of freedom. These conservative contrast intervals and the design-degree-of-freedom coefficient intervals address different inferential summaries; their rationale is specified in Supplementary Methods M3. Analyses were descriptive, without multiplicity correction (Supplementary Tables S2–S3; Fig. S1). Analyses used R 4.5.3 and the survey package (Lumley, 2004; R Core Team, 2026).

### 2.7 Ethics and protection of participants

NHANES received approval from the National Center for Health Statistics Ethics Review Board and obtained participant consent (Terry et al., 2024). The corresponding author's institution granted an exemption from ethics review for this secondary analysis of public, deidentified survey data. Analyses involved no participant contact or reidentification and reported aggregate results to protect participant safety and privacy.

## 3 Results

### 3.1 National CKM stage distribution

The NHANES sample included 5006 eligible adults. Stage 2 was most common at 45.8% (95% CI, 43.8%, 47.8%); stages 0, 1, 3, and 4 represented 13.1%, 28.1%, 4.0%, and 8.9%, respectively (Table 1; Fig. 1A). Four participants (<0.1% weighted) remained unresolved.

**Table 1:** Cardiovascular-kidney-metabolic syndrome stages among United States adults aged ≥20 years, National Health and Nutrition Examination Survey, August 2021–August 2023.

Syndrome stage	Unweighted n	Weighted % (95% confidence interval)
Stage 0	543	13.1 (11.1, 15.4)
Stage 1	1196	28.1 (25.8, 30.5)
Stage 2	2368	45.8 (43.8, 47.8)
Stage 3	294	4.0 (3.4, 4.8)
Stage 4	601	8.9 (7.8, 10.1)
Unresolved	4	<0.1 (<0.1, 0.1)
Stages 3–4	895	13.0 (11.6, 14.4)

Values are survey-weighted percentages with 95% confidence intervals.

Denominator: 5006 adults, including four with unresolved risk-based staging. Percentages and Korn–Graubard beta intervals use phlebotomy survey weights. Stages 3–4 form a subtotal. Unresolved participants are retained separately; “<0.1” indicates a positive value below the displayed precision. Counts are unweighted.

**Fig. 1:**
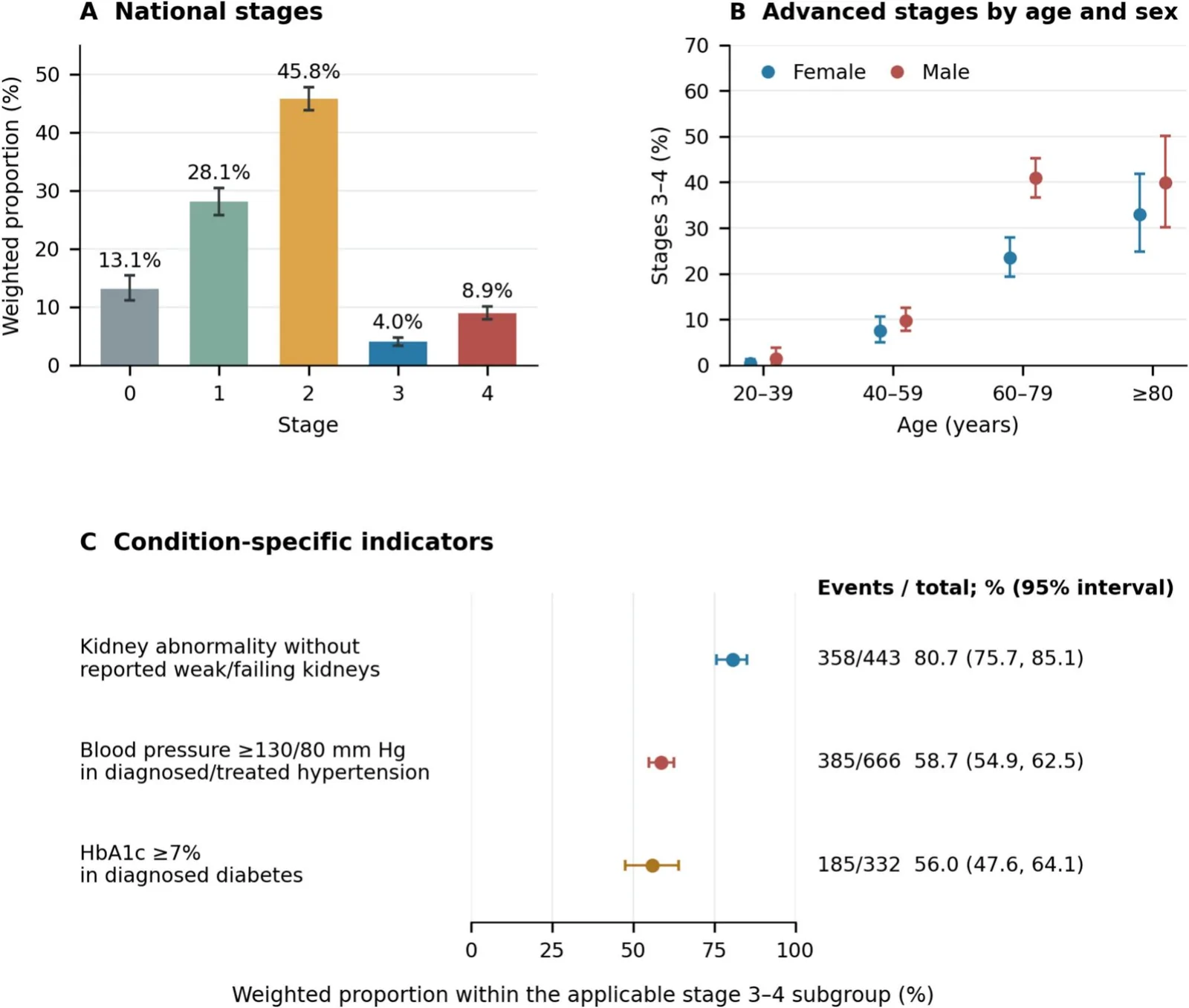
Cardiovascular-kidney-metabolic syndrome stages and clinical indicators among United States adults aged ≥20 years, National Health and Nutrition Examination Survey, August 2021–August 2023. A: National stages (5006 adults). B: Stages 3–4 by age and sex; groups describe cross-sectional membership. C: Observed-response clinical subgroups defined in Table 2. Intervals are survey-weighted Korn–Graubard 95% confidence intervals. Counts are unweighted. HbA1c denotes glycated hemoglobin; four unresolved stages remain in the national denominator.

Advanced CKM affected 13.0% (95% CI, 11.6%, 14.4%) of adults and was more common in older adults (Fig. 1B). Prevalence was 13.9% (95% CI, 12.4%, 15.5%) with selection weighting. Results under matched fasting and alternative PREVENT definitions are reported in Supplementary Table S2.

### 3.2 Recognition, control, access, and social-risk indicators among adults with advanced CKM

Among adults with advanced CKM and a single-visit kidney abnormality, 80.7% (95% CI, 75.7%, 85.1%) did not report weak or failing kidneys (Table 2; Fig. 1C). Of those with diagnosed or treated hypertension, 58.7% (95% CI, 54.9%, 62.5%) had blood pressure ≥ 130/80 mm Hg; of those with diagnosed diabetes, 56.0% (95% CI, 47.6%, 64.1%) had HbA1c ≥7%. Lipid-lowering medication was not reported by 18.6% (95% CI, 14.7%, 23.0%) of the high-cholesterol proxy group. Low or very low adult food security was present in 18.8% (95% CI, 14.8%, 23.2%) of those with observed responses. No usual place for care and no health insurance were reported by 4.0% and 3.3%, respectively. Higher-threshold and IPW results appear in Supplementary Table S2.

**Table 2:** Recognition, clinical and social indicators among United States adults with advanced cardiovascular-kidney-metabolic syndrome, National Health and Nutrition Examination Survey, August 2021–August 2023.

Indicator	Events / total	Weighted % (95% confidence interval)
Kidney abnormality without reported weak/failing kidneys	358/443	80.7 (75.7, 85.1)
Blood pressure ≥ 130/80 mm Hg	385/666	58.7 (54.9, 62.5)
Glycated hemoglobin ≥7%	185/332	56.0 (47.6, 64.1)
No reported lipid-lowering medication	115/621	18.6 (14.7, 23.0)
Low/very low food security among household adults	164/834	18.8 (14.8, 23.2)
No usual place for care	33/895	4.0 (2.3, 6.2)
No health insurance	27/895	3.3 (2.0, 5.1)
Both blood pressure ≥ 130/80 mm Hg and glycated hemoglobin ≥7%	91/277	34.1 (24.7, 44.4)

Intervals are 95% confidence intervals; event counts and totals are unweighted.

All rows require stages 3–4 and an observed response. Blood pressure and glycemia use diagnosed/treated hypertension and diagnosed diabetes, respectively; the joint row requires both conditions and both thresholds. Kidney abnormality is single-visit estimated glomerular filtration rate < 60 mL/min/1.73 m^2^ or urine albumin-to-creatinine ratio ≥ 30 mg/g. The lipid denominator is total cholesterol ≥240 mg/dL or reported high cholesterol. Two unknown kidney responses and 61 missing food-security responses are excluded. Percentages and Korn–Graubard intervals are survey weighted. These markers do not establish persistent kidney disease or individual treatment failure.

In the fixed subgroup with diagnosed/treated hypertension and diagnosed diabetes, 91/277 had both blood pressure ≥ 130/80 mm Hg and HbA1c ≥7%: 34.1% (95% CI, 24.7%, 44.4%). At blood pressure ≥ 140/90 mm Hg and HbA1c ≥8%, 32/277 met both criteria: 12.6% (95% CI, 7.3%, 19.9%). Exact-stage and sensitivity results are secondary (Supplementary Table S2; Fig. S1).

### 3.3 Exploratory state-level profiles

The CVD-free training sample contained 4395 participants and 293 stage-3 cases. After excluding 601 stage-4 participants, four had unresolved staging and six additional participants lacked a predictor, including one stage-3 case (Supplementary Table S1). Cross-validated discrimination, calibration and bootstrap-corrected performance are shown in Supplementary Fig. S2. BRFSS excluded 4718 respondents with unknown common CVD (1.1% weighted). Among 366,062 with negative common CVD history, 343,715 had complete predictors (92.8% weighted). The largest IPW-versus-complete-case change in the crude modeled contribution was <0.1 percentage points.

Across 49 jurisdictions, reported common-CVD prevalence ranged from 5.5% to 14.7% crude and 6.8% to 13.5% age standardized. The modeled stage-3 contribution ranged from 3.4% to 6.8% crude and 4.4% to 6.5% age standardized (Fig. 2; Supplementary Table S4). These components are conditional exploratory outputs, not estimates of full state advanced-CKM prevalence.

**Fig. 2:**
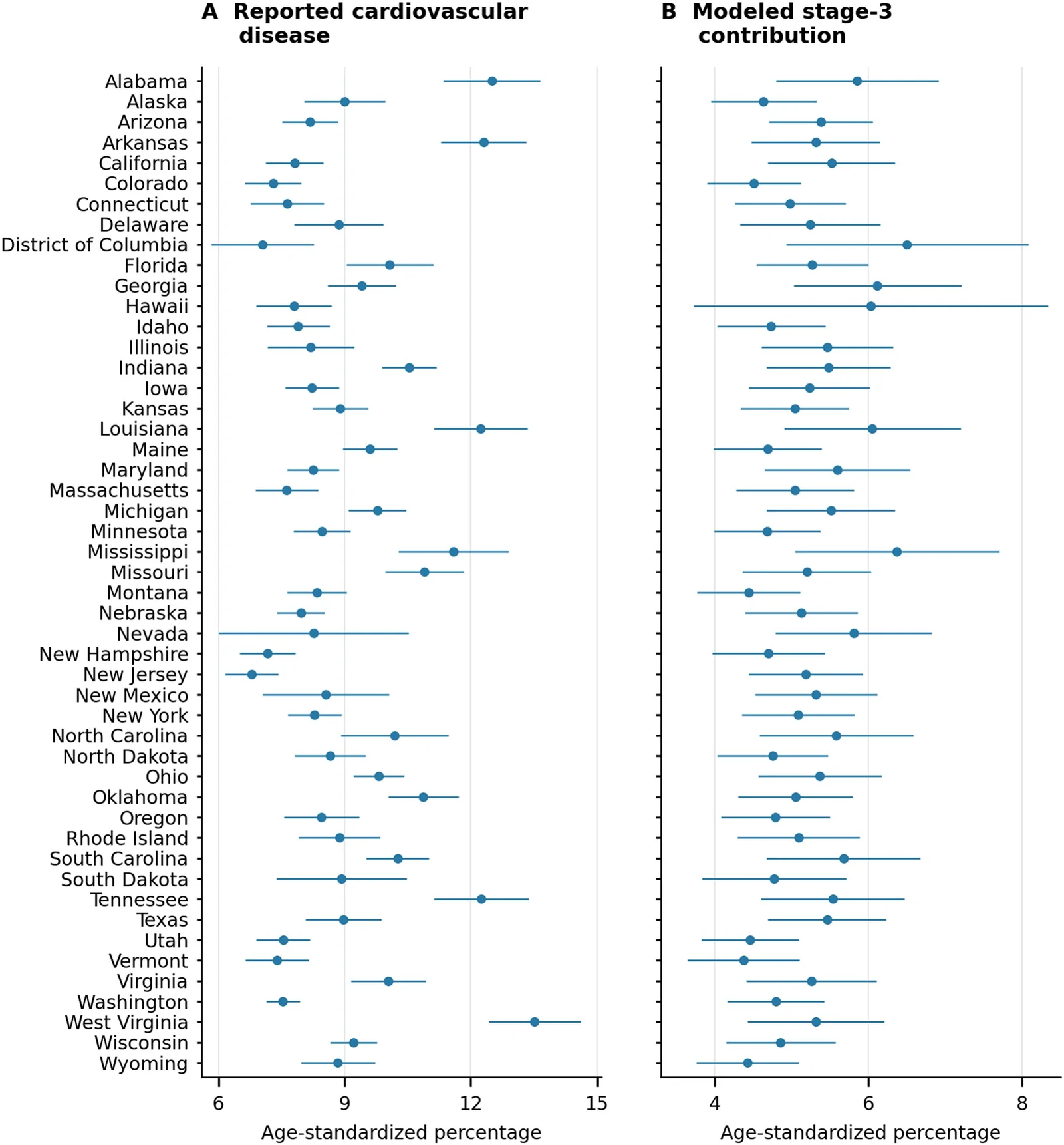
Separate reported cardiovascular disease and modeled stage-3 components among adults aged ≥20 years in 48 states and the District of Columbia, United States, Behavioral Risk Factor Surveillance System, 2023. A: Reported myocardial infarction, coronary disease/angina or stroke. B: Stage-3 contribution from the 2021–2023 national survey model. Both condition on known cardiovascular disease status and use the fixed pooled age reference. Bars show conditional delta-method 95% confidence intervals. Components are separate and do not sum to full advanced syndrome prevalence. Kentucky and Pennsylvania were unavailable. Table S4 and Methods M4–M5 give denominators, weighting and excluded uncertainties.

Model specification affected geographic patterns: the demographic-only model differed from the primary model by up to 1.5 percentage points in the standardized stage-3 contribution (Spearman correlation, 0.64); the model excluding kidney report differed by up to 0.2 points (Supplementary Fig. S2). Contextual indicators retain their source-specific units and periods (Supplementary Table S4).

### 3.4 Socioeconomic gradients

Lower education and income were associated with higher advanced CKM prevalence in both demographic-adjusted and full models, with different magnitudes. Insurance and food-security associations varied with adjustment. Paired estimates and model diagnostics appear in Supplementary Table S3.

## 4 Discussion

Among adults with advanced CKM and a single-visit kidney abnormality, 80.7% did not report weak or failing kidneys. Elevated blood pressure and HbA1c were common in their respective clinical subgroups. Approximately one third of adults with advanced CKM, diagnosed or treated hypertension, and diagnosed diabetes had elevations in both measures. Advanced CKM affected about one in eight United States adults, while stage 2 accounted for nearly half. Exploratory state profiles separated directly reported CVD, modeled stage-3 contribution, and contextual descriptors.

These findings build on established evidence of CKM prevalence, multimorbidity, and inadequate risk-factor control. Ostrominski et al. (2025) examined comprehensive control in adults with at least two CKM conditions. Lee et al. (2025) assessed joint control in hypertension with concurrent diabetes or hyperlipidemia, including the 2021–2023 cycle. Gong et al. (2026a) assessed treatment rates in CKM stage ≥2 and risk-factor control among treated adults, including risk and condition-count strata. We describe kidney reporting and clinical indicators among adults with advanced CKM and quantify simultaneous blood pressure and glycemic elevation in those with diagnosed or treated hypertension and diagnosed diabetes. Differences in eligibility, thresholds, and standardization preclude direct numerical comparisons. Joint elevation is also distinct from failure to achieve control of all conditions: a person with only one elevated measure meets the latter but not the former.

Stage 2 represents a broad upstream population for risk-factor prevention. Within advanced CKM, condition-specific denominators distinguish kidney reporting, blood pressure and glycemic measurements, and lipid-medication reports. The observed patterns are consistent with national reports (Fryar et al., 2024; Gong et al., 2026b; Inoue et al., 2025). Yim et al. (2025) provide related international context on CKM trends and socioeconomic disparities in South Korea.

Routine eGFR and UACR testing can identify abnormalities that warrant confirmatory evaluation, consistent with kidney-disease guidance (Kidney Disease: Improving Global Outcomes (KDIGO) CKD Work Group, 2024). Standardized blood pressure protocols, medication review, home monitoring, and team-based follow-up are established program components (Wall et al., 2023), but this study did not evaluate their effectiveness or delivery. Food insecurity, insurance, and usual-care indicators provide context for surveillance; they do not measure local implementation capacity.

Stage-specific interpretation is constrained by the staging definition. PREVENT incorporates blood pressure, diabetes, eGFR, and optional HbA1c/UACR, so selection into stage 3 enriches several markers being described. A fixed hypertension-plus-diabetes subgroup does not remove this dependence. Omitting optional inputs reduced stage-3 HbA1c elevation from 65.3% to 52.6%; stage 4 remained at 50.1%. After restricting the comparison to adults aged 45–79 in sex-by-race/ethnicity groups represented in both stages, the adjusted difference in joint elevation (stage 3 minus stage 4) was 17.83 percentage points (95% CI, −0.22, 35.87). These descriptive comparisons therefore provide limited evidence about independent management-quality differences.

The state profiles extend the geographic description of CKM-related burden through separate reported-CVD and modeled stage-3 components. They do not estimate state-specific kidney nonreporting, blood pressure control, or glycemic control. Cardiometabolic and social/access descriptors provide context in their source units. Their different age ranges, source years, and measurement methods limit direct comparison; some also draw on BRFSS and cannot independently validate the bridge.

Socioeconomic findings describe the context in which these clinical indicators occur (Havranek et al., 2015; National Academies of Sciences, Engineering, and Medicine, 2017). Education and income associations were directionally consistent across the reported models, whereas insurance and food-security associations depended on specification. Limited PSUs constrain model inference, and the full model does not support a joint test of all coefficients. The models are descriptive, differ in age specification and adjustment, and do not establish causal effects. The inverse association between being uninsured and advanced CKM should not be interpreted as a protective effect of lacking insurance.

Several measurement limitations warrant emphasis. Operational CKM staging cannot reproduce a full clinical assessment. Single-visit eGFR and UACR cannot establish persistent CKD, and KIQ022 asks about weak or failing kidneys rather than all CKD diagnoses. Nonreporting may therefore reflect question wording or communication as well as lack of recognition. Blood pressure and HbA1c thresholds are fixed surveillance markers; the study cannot determine whether each value exceeds an appropriate individual target. Detailed medication and low-density lipoprotein cholesterol information was not incorporated in the primary analytic dataset, limiting the lipid indicator to medication reporting. Access and food-security reports also do not measure the quality or adequacy of care.

State projections depend on the transportability of operational stage-3 relationships, including their strong age structure. BRFSS does not capture heart failure, so its common-CVD-negative population differs from the training population. The model has 12 slopes, 15 survey design degrees of freedom, and three residual degrees of freedom. Internal validation cannot substitute for external measured-state validation. The normal interval approximation is conditional; finite-degree-of-freedom alternatives widen intervals, and neither captures structural transport error or response-weight estimation uncertainty.

Selection and classification also limit generalizability. Complete cases represented 87.1% of weighted phlebotomy eligibility; low survey response and indicator-specific missing responses may produce additional selection. IPW addresses observed analytic inclusion under its response-model assumptions, not survey nonparticipation or unmeasured nonrandom selection. The PREVENT boundary convention approximates risk while preserving clinical measurements and can alter stage membership. Outside ages 30–79, risk-based stage 3 is not assigned, potentially undercapturing stage 3 among older adults. Finally, the small fixed subgroup, particularly stage 3, limits precision, and all revision-stage analyses are exploratory.

These cross-sectional data cannot establish causal effects or whether specific programs improve outcomes. Local measured-data validation and assessment of service capacity are required before using the state projections in planning.

## 5 Conclusions

Advanced CKM affected about one in eight United States adults, with frequent kidney nonreporting and condition-specific clinical abnormalities. Approximately one third of adults with both hypertension and diabetes in this group had simultaneous blood pressure and glycemic elevation. These national findings and the separate exploratory geographic profiles provide complementary information for surveillance and hypothesis generation.

## Declaration of generative artificial intelligence use

ChatGPT™ (version 5.6 Sol) was used for code review and language editing. The authors reviewed the outputs and take responsibility for the final manuscript.

## CRediT authorship contribution statement

**Menghao Zhou:** Writing – original draft, Visualization, Methodology, Formal analysis, Conceptualization. **Fen Zhang:** Writing – review & editing, Validation. **Bingluo Zhou:** Writing – review & editing, Validation. **Yiming Lv:** Writing – review & editing, Supervision, Conceptualization.

## Ethics declaration

### Informed consent and patient details

Written informed consent to take part in the study and to publish the article has been obtained from all participants or their legal representatives. The privacy rights of participants have been observed.

### Studies in human

This study was performed in compliance with relevant laws, regulatory frameworks and guidelines where the research took place. The Ethics Committee of Sir Run Run Shaw Hospital, Zhejiang University School of Medicine granted an exemption. The authors provided the following explanation: This study was exempted from further ethical review by the Ethics Committee of Sir Run Run Shaw Hospital, Zhejiang University School of Medicine because it involved only secondary analysis of publicly available, de-identified data from the National Health and Nutrition Examination Survey (NHANES), with no direct participant contact or access to identifiable private information. The original NHANES protocols were approved by the National Center for Health Statistics (NCHS) Ethics Review Board, and written informed consent was obtained from all participants or their legal representatives, as applicable.

## Funding

This research received no funding.

## Declaration of competing interest

The authors declare that they have no known competing financial interests or personal relationships that could have appeared to influence the work reported in this paper.

## Data Availability

Public survey files are available from NHANES and BRFSS. Supplementary_Source_Data.xlsx documents the sources and provides numerical results, including kidney-definition sensitivities. Final analysis code is publicly available on GitHub™ (version 5c9e311; https://github.com/Elliott0114/pmedr-ckm-analysis/tree/5c9e3116ae8c7140ac3a20d14d0f27fdfd5fdb80). The scripts rebuild the national analyses and exploratory state projections from the public survey files using the documented input files and locked package versions.

## Appendix A Supplementary data

Supplementary material 1 Supplementary Methods M1–M6, Tables S1–S4, and Figures S1–S2: operational definitions, statistical methods, sensitivity analyses, and exploratory state-level analyses.

Supplementary material 2 Supplementary source data: data-source documentation and numerical results for national analyses, kidney-definition sensitivity analyses, and exploratory state-level analyses.

## References

[bb0005] Aggarwal R., Ostrominski J.W., Vaduganathan M. (2024). Prevalence of cardiovascular-kidney-metabolic syndrome stages in US adults, 2011-2020. JAMA.

[bb0010] American Heart Association (2026). PREVENT frequently asked questions. https://professional.heart.org/-/media/PHD-Files/Guidelines-and-Statements/PREVENT/PREVENT-FAQs-FINAL.pdf?sc_lang=en.

[bb0015] Centers for Disease Control and Prevention (2025). 2023 BRFSS survey data and documentation. https://www.cdc.gov/brfss/annual_data/annual_2023.html.

[bb0020] Centers for Disease Control and Prevention (2024). BRFSS combined landline and cell phone weighted response rates by state, 2023. https://www.cdc.gov/brfss/annual_data/2023/pdf/2023_ResponseRates_Table-508.pdf.

[bb0025] Chang R., Parekh T., Hagan K.K., Javed Z., Ostrominski J.W. (2025). State-level prevalence of cardiovascular-kidney-metabolic syndrome stages in the United States, 2011 to 2023. JACC: Advances.

[bb0030] Flanagan B.E., Gregory E.W., Hallisey E.J., Heitgerd J.L., Lewis B. (2011). A social vulnerability index for disaster management. Journal of Homeland Security and Emergency Management.

[bb0035] Fryar C.D., Kit B.K., Carroll M.D., Afful J. (2024). Hypertension prevalence, awareness, treatment, and control among adults age 18 and older: United States, august 2021-august 2023. NCHS Data Brief.

[bb0040] Gong J., Chiu N., Clinch K.A., Qian Y., Ho J.E., Wadhera R.K. (2026). Treatment of cardiometabolic risk factors among U.S. adults with cardiovascular-kidney-metabolic syndrome. J. Am. Coll. Cardiol..

[bb0045] Gong J., Vaduganathan M., Wadhera R.K. (2026). Chronic kidney disease prevalence and awareness among US adults. JAMA Cardiol..

[bb0050] Greenlund K.J., Lu H., Wang Y., Matthews K.A., LeClercq J.M., Lee B., Carlson S.A. (2022). PLACES: local data for better health. Prev. Chronic Dis..

[bb0055] Havranek E.P., Mujahid M.S., Barr D.A., Blair I.V., Cohen M.S., Cruz-Flores S., Davey-Smith G., Dennison-Himmelfarb C.R., Lauer M.S., Lockwood D.W., Rosal M., Yancy C.W. (2015). Social determinants of risk and outcomes for cardiovascular disease. Circulation.

[bb0060] Health Resources and Services Administration (2025). National Center for Health Workforce Analysis. (2025). State of the primary care workforce. https://bhw.hrsa.gov/sites/default/files/bureau-health-workforce/data-research/State-of-the-Primary-Care-Workforce-2025.pdf.

[bb0065] Inker L.A., Eneanya N.D., Coresh J., Tighiouart H., Wang D., Sang Y., Crews D.C., Doria A., Estrella M.M., Froissart M., Grams M.E., Greene T., Grubb A., Gudnason V., Gutiérrez O.M., Kalil R., Karger A.B., Mauer M., Navis G. (2021). New creatinine- and cystatin C–based equations to estimate GFR without race. N. Engl. J. Med..

[bb0070] Inoue K., Liu M., Aggarwal R., Marinacci L.X., Wadhera R.K. (2025). Prevalence and control of diabetes among US adults, 2013 to 2023. JAMA.

[bb0075] Khan S.S., Matsushita K., Sang Y., Ballew S.H., Grams M.E., Surapaneni A., Blaha M.J., Carson A.P., Chang A.R., Ciemins E., Go A.S., Gutierrez O.M., Hwang S.-J., Jassal S.K., Kovesdy C.P., Lloyd-Jones D.M., Shlipak M.G., Palaniappan L.P., Sperling L. (2024). Development and validation of the American Heart Association’s PREVENT equations. Circulation.

[bb0080] Kidney Disease: Improving Global Outcomes (KDIGO) CKD Work Group (2024). KDIGO 2024 clinical practice guideline for the evaluation and management of chronic kidney disease. Kidney Int..

[bb0085] Krishnan V., Huang X., McGowan C., Shah N.S., Qureshi F., Minkovitz C.S., Kershaw K.N., Freedman A.A., Miller G.E., Lloyd-Jones D.M., Khan S.S. (2026). Neighborhood indices, income, and cardiovascular-kidney-metabolic syndrome at the census tract level. JAMA Netw. Open.

[bb0090] Lee H.-H., Crawford E.B., Cho S.M.J., Krawisz A.K., Juraschek S.P., Cluett J.L., Lee H., Kim H.C., Natarajan P., Wadhera R.K., Secemsky E.A. (2025). Trends in prevalence, treatment, and control of cardiometabolic risk factors among adults with hypertension in the United States, 1999–2023. J. Am. Coll. Cardiol..

[bb0095] Li J., Lei L., Wang W., Ding W., Yu Y., Pu B., Peng Y., Li Y., Zhang L., Guo Y. (2024). Social risk profile and cardiovascular-kidney-metabolic syndrome in US adults. J. Am. Heart Assoc..

[bb0100] Lumley T. (2004). Analysis of complex survey samples. J. Stat. Softw..

[bb0105] Minhas A.M.K., Mathew R.O., Sperling L.S., Nambi V., Virani S.S., Navaneethan S.D., Shapiro M.D., Abramov D. (2024). Prevalence of the cardiovascular-kidney-metabolic syndrome in the United States. J. Am. Coll. Cardiol..

[bb0110] National Academies of Sciences, Engineering, and Medicine (2017).

[bb0115] Ndumele C.E., Rangaswami J., Chow S.L., Neeland I.J., Tuttle K.R., Khan S.S., Coresh J., Mathew R.O., Baker-Smith C.M., Carnethon M.R., Despres J.-P., Ho J.E., Joseph J.J., Kernan W.N., Khera A., Kosiborod M.N., Lekavich C.L., Lewis E.F., Lo K.B. (2023). Cardiovascular-kidney-metabolic health: a presidential advisory from the American heart association. Circulation.

[bb0120] Ndumele C.E., Rodriguez F., Dixon D.L., Khan S.S., Mukherjee D., Bajaj M., Bangalore S., Bozkurt B., Breathett K., Clarke S.L., de Boer I.H., Ellison D.H., Evangelista L.S., Heffron S.P., Kazi D.S., Kulshreshtha A., Lingvay I., Low Wang C.C., Mercado C.A. (2026). 2026 AHA/ACC/ADA/ASN guideline for the prevention, detection, evaluation, and management of cardiovascular-kidney-metabolic syndrome. J. Am. Coll. Cardiol..

[bb0125] Ostrominski J.W., Hirsch J.S., Miao B., Clark B., Donato B.M.K., Woolley J.J., Parrinello C.M., O’Connell T., Vaduganathan M. (2025). Risk factor control in U.S. adults with cardiovascular-kidney-metabolic multimorbidity, 1999–2020. J. Am. Coll. Cardiol..

[bb0130] Parker J.D., Talih M., Malec D.J. (2017). National Center for Health Statistics data presentation standards for proportions. Vital and Health Statistics, Series 2, No. 175. https://www.cdc.gov/nchs/data/series/sr_02/sr02_175.pdf.

[bb0135] R Core Team (2026). https://www.R-project.org/.

[bb0140] Rabbitt M.P., Reed-Jones M., Hales L.J., Burke M.P., U.S. Department of Agriculture, Economic Research Service (2024). Household food security in the United States in 2023 (economic research report no. 337).

[bb0145] Terry A.L., Chiappa M., McAllister J., Woodwell D., Graber J., National Center for Health Statistics (2024). Plan and operations of the National health and nutrition examination survey, august 2021-august 2023 (vital and health statistics, series 1, no. 66).

[bb0150] Wall H.K., Wright J.S., Fleisher L.A., Sperling L.S. (2023). Improving blood pressure control in the United States: at the heart of million hearts 2027. Am. J. Hypertens..

[bb0155] Yim Y., Lee J.E., Son Y. (2025). Long-term trends in the prevalence of cardiovascular-kidney-metabolic syndrome in South Korea, 2011–2021: a representative longitudinal serial study. The Lancet Regional Health – Western Pacific.

[bb0160] Zhu R., Wang R., He J., Wang L., Chen H., Niu X., Sun Y., Guan Y., Gong Y., Zhang L., An P., Li K., Ren F., Xu W., Guo J. (2024). Prevalence of cardiovascular-kidney-metabolic syndrome stages by social determinants of health. JAMA Netw. Open.

