# Supplementary material for: Recognition and control gaps in advanced cardiovascular-kidney-metabolic syndrome among United States adults
Source: Prev Med Rep. 2026 Sep 20;71:103635. doi: 10.1016/j.pmedr.2026.103635 (PMC13633931; doi:10.1016/j.pmedr.2026.103635)
Supplement: Supplementary file 1 [file mmc1.docx]

Supplementary Material

Recognition and Control Gaps in Advanced Cardiovascular-Kidney-Metabolic Syndrome Among United States Adults

Four supplementary tables and two supplementary figures. Methods precede the displays. Supplementary_Source_Data.xlsx contains source documentation and numerical results. Final analysis code is publicly available on GitHub™ (version 5c9e311; https://github.com/Elliott0114/pmedr-ckm-analysis/tree/5c9e3116ae8c7140ac3a20d14d0f27fdfd5fdb80). The repository includes the source-file list, locked package versions and scripts for rebuilding the final national and exploratory state analyses from the public survey files.

## Supplementary Methods

Abbreviations used in these methods: cardiovascular-kidney-metabolic (CKM); cardiovascular disease (CVD); chronic kidney disease (CKD); National Health and Nutrition Examination Survey (NHANES); Behavioral Risk Factor Surveillance System (BRFSS); glycated hemoglobin (HbA1c); estimated glomerular filtration rate (eGFR); urine albumin-to-creatinine ratio (UACR); Predicting Risk of Cardiovascular Disease Events (PREVENT); inverse-probability weighting (IPW); primary sampling unit (PSU); poverty income ratio (PIR); confidence interval (CI); National Center for Health Statistics (NCHS).

## M1. Operational definitions and clinical denominators

Stage hierarchy. Assign the highest captured stage. Stage 4 overrides stage 3; stage 3 overrides stage 2; stage 2 overrides stage 1. Four unresolved risks remain separate.

Stage 0 / Stage 1. No captured risk factors / body mass index ≥25 kg/m² or waist >102 cm (men), >88 cm (women), or prediabetes. Prediabetes: HbA1c 5.7–<6.5%, reported prediabetes, or borderline diabetes without captured diabetes.

Stage 2. Hypertension, diabetes, kidney abnormality, or ≥3 of four nonfasting metabolic proxy components: abdominal adiposity, hypertension, dysglycemia, low high-density lipoprotein cholesterol. This is not the full triglyceride-based metabolic syndrome definition.

Hypertension / diabetes for staging. Hypertension: measured blood pressure ≥130/80 or reported hypertension or antihypertensive use. Diabetes: HbA1c ≥6.5%, diagnosed diabetes, or reported glucose-lowering medication.

Very-high-risk kidney criterion. eGFR <30; or eGFR 30–<45 with UACR ≥30; or eGFR 45–<60 with UACR ≥300. Units: mL/min/1.73 m² and mg/g. Single-visit proxy, not confirmed chronic disease.

Stage 3 / Stage 4. Very-high-risk kidney criterion or 10-year PREVENT total CVD risk ≥20% without captured CVD / report of heart failure, coronary heart disease, angina, myocardial infarction, or stroke (MCQ160B–F).

PREVENT model. preventr 0.12.0 total-CVD equations, 10 years, ages 30–79, no captured CVD. Automatic optional-model selection uses HbA1c and UACR when valid; no social deprivation index was supplied. Thus the analysis is not a uniform base-equation analysis. Lipid-lowering medication report is a statin-use proxy.

Required input bounds. Systolic blood pressure 90–200 mm Hg; total cholesterol 130–320 mg/dL; high-density lipoprotein cholesterol 20–100 mg/dL; eGFR 15–140; body mass index 18.5–39.9 kg/m². Only risk-calculation copies are bounded. Age is never clipped to create eligibility.

Optional input handling. Observed HbA1c and UACR are supplied; invalid optional values trigger the package’s compatible-model selection, rather than modification of the original biomarker. HbA1c 3–15% accepted under the American Heart Association range.

Age / data limits. Outside 30–79, stage 3 can arise only from the kidney criterion; subclinical imaging and uncaptured clinical CVD remain unavailable. Missing required risk inputs without kidney-stage-3 evidence are unresolved, not negative.

Kidney reporting marker. eGFR <60 or UACR ≥30, combined with KIQ022 = 2 (no report of being told of weak or failing kidneys). KIQ022 = 1 means yes; refused/don’t-know responses are missing.

Blood pressure and HbA1c denominators. Blood pressure: hypertension report or treatment. HbA1c: diagnosed diabetes (DIQ010 = 1). A fixed dual-condition denominator requires both. Marker analyses also require the relevant observed measurement/response.

Local PREVENT adapter. The installed preventr 0.12.0 implementation uses discretionary systolic blood pressure and HbA1c limits of 180 mm Hg and 4.5%, whereas its documentation identifies equation limits of 200 mm Hg and 3%. A local scalar adapter changes these two input validators; equation coefficients and the bodies of the risk and model-selection functions are unchanged. Common-range predictions matched the installed package over 36 test combinations. The adapter is provided in R/survey_estimators.R in the public code repository; verification results are in Supplementary_Source_Data.xlsx, sheet Additional_outputs, dataset new_adapter_stage_checks.csv. Age is not clipped to establish PREVENT eligibility.

Lipid and social indicators. The lipid denominator comprises total cholesterol ≥240 mg/dL or a reported high-cholesterol diagnosis; the event is no reported lipid-lowering medication. Low/very low adult food security is FSDAD 3/4; codes 1/2 are full/marginal security and other/missing responses are excluded. No usual place for care and no health insurance use observed survey responses. These measures have separate eligible and observed denominators (Table S1). International-system equivalents of conventional units: blood pressure 130/80 and 140/90 mm Hg correspond to 17.3/10.7 and 18.7/12.0 kPa; cholesterol 240 mg/dL corresponds to 6.2 mmol/L. For other thresholds, multiply cholesterol in mg/dL by 0.02586, triglycerides by 0.01129, and glucose by 0.05551 to obtain mmol/L; multiply creatinine in mg/dL by 88.4 to obtain micromol/L and urine albumin-to-creatinine ratio in mg/g by 0.11312 to obtain mg/mmol. Conversions describe units only; the original measurement units and thresholds were used in every analysis.

Matched fasting sensitivity. The same 2,592 fasting participants and WTSAF2YR weights are used for both definitions. The augmented definition adds fasting glucose ≥126 mg/dL for diabetes, 100–<126 mg/dL for prediabetes, and triglycerides ≥150 mg/dL within the metabolic-risk definition. Fasting-defined diabetes is passed to PREVENT. There are 47 additional diabetes classifications and 200 stage changes; one risk remains unresolved. This revision-stage sensitivity assesses the definition within the fasting sample, not an unweighted comparison with the full phlebotomy sample.

## M2. Analytic selection, proportion intervals and reliability

NHANES completeness. Age, sex, body mass index, waist, mean systolic and diastolic blood pressure, HbA1c, total and high-density lipoprotein cholesterol, creatinine/eGFR, UACR, hypertension report, antihypertensive use, lipid medication proxy, full captured CVD history. Additional risk inputs are checked separately.

NHANES IPW was re-specified and refitted during revision. The submitted implementation used a four-degree-of-freedom age spline, sex, race/ethnicity, education, PIR with a missingness indicator, and insurance, with fixed probability truncation at 0.05–0.99. The revision model uses a three-degree-of-freedom age spline and additionally includes reported hypertension, diabetes, weak/failing kidneys, captured CVD, and smoking. This is a revision-stage sensitivity-design change, not a correction of manuscript wording alone and not a consequence of CKM reclassification alone.

Weighted quasibinomial logistic model: natural age spline (3 degrees of freedom), sex, six race/ethnicity categories, education, poverty-income ratio with missingness indicator, insurance, reported hypertension, diabetes, weak/failing kidneys, full captured CVD, and smoking. Missing categorical predictors receive a missing level; PIR is median-filled with a missing indicator. The complete-case first and 99th percentiles determined the probability truncation limits; the calculation and implementation guardrails are provided in R/national_selection.R in the public code repository. Phlebotomy weights are divided by the truncated probability. This approach addresses observed analytic completeness under model assumptions; it does not correct survey nonparticipation, unknown endpoint responses, or unmeasured nonrandom selection. Response-model estimation uncertainty is not propagated.

Proportion intervals. Let p denote the survey proportion, V its design variance, n its observed denominator, and f the number of represented PSUs minus represented strata. The reported effective size is min(n, p(1−p)/V). The Korn–Graubard beta calculation uses n*=min[n, {p(1−p)/V}{t(0.975,n−1)/t(0.975,f)}²], x*=p n*, and beta quantiles B0.025(x*,n*−x*+1) and B0.975(x*+1,n*−x*). The F-quantile identity independently checks implementation. Changing the interval method leaves weighted point estimates unchanged (Parker et al., 2017).

Reliability. A dagger denotes n<30, effective n<30, interval width ≥30 percentage points, or width between 5 and 30 points with relative width >130%; design degrees of freedom<8 also requires review. For p>0.5, a failing complementary proportion is flagged as a caution rather than automatically suppressed. Boundary proportions and nonestimable intervals retain their counts and are labelled without a CI. Full numerical checks, including Wald limits, effective sizes, degrees of freedom and flags, remain in the Excel source-data workbook; flags are not a certification of NCHS publication eligibility.

## M3. Secondary adjusted comparisons and inferential choices

Stage comparisons use survey logistic regression adjusted for age, sex and race/ethnicity. The pooled target is the applicable stage 2–4 population. The support sensitivity restricts stages 3–4 to ages 45–79 and sex-by-race/ethnicity cells represented in both stages, then standardizes to that restricted applicable population. These targets differ; their contrast is not a formal test of effect modification. Each contrast uses its model residual degrees of freedom (6 for the pooled and 7 for the restricted models shown). Failed fits, nonestimable parameters, insufficient covariance rank or nonpositive residual degrees of freedom are not rescued by variable deletion.

For individual equity coefficients, both the full and demographic-plus-one-factor models consistently use t(15), the design-degree-of-freedom approximation. The survey documentation permits design-degree-of-freedom inference and notes that subtracting all coefficients can be conservative for person-level covariates. We nevertheless retain the more conservative residual-degree-of-freedom rule for the nonlinear stage-standardized contrasts, whose sparse stage-specific support warrants caution. These are explicit, different inferential conventions, not choices made separately according to significance. Neither rule removes limited-PSU uncertainty.

The equity models use the same 4,382 complete cases, including 770 advanced-CKM cases. The full model includes categorical age, sex, race/ethnicity and all four social factors; comparison models use a natural age spline (2 degrees of freedom), sex, race/ethnicity and one social factor. Thus both age specification and mutual social adjustment differ. The full model is a supplementary, more extensively adjusted description. Its 16 slopes exceed the 15 design degrees of freedom, and its covariance rank is 15; no full-parameter joint Wald test is performed. Individual coefficient intervals are approximate and do not repair a nonidentifiable omnibus test. Interpretive weight rests on consistency and differences across the paired specifications, not model selection by significance.

## M4. State-model harmonization and internal diagnostics

Age. NHANES: RIDAGEYR. BRFSS / application: _AGE80, top-coded at 80; natural spline degrees of freedom=2 plus ages-30–79 indicator.

Sex. NHANES: RIAGENDR. BRFSS / application: SEXVAR; female/male as recorded.

Race/ethnicity. NHANES: RIDRETH3; Hispanic categories combined for bridge. BRFSS / application: _IMPRACE; non-Hispanic White, Black, Asian, Hispanic, other/multiracial.

Hypertension. NHANES: BPQ020, yes/no. BRFSS / application: BPHIGH6: 1 yes; 2/3/4 no; other missing.

Diabetes. NHANES: DIQ010 = 1. BRFSS / application: DIABETE4: 1 yes; 2/3/4 no; other missing.

Kidney report. NHANES: KIQ022: weak/failing kidneys. BRFSS / application: CHCKDNY2: kidney disease report; question wording differs.

Smoking. NHANES: SMQ020 + SMQ040. BRFSS / application: _SMOKER3: 1/2 current, 3/4 not current.

CVD exclusion / direct component. NHANES: No heart failure, coronary disease, angina, myocardial infarction, or stroke; all five histories resolved. BRFSS / application: Direct component: CVDINFR4, CVDCRHD4, CVDSTRK3; any yes / all no / otherwise missing. Heart failure is unavailable.

Primary model. NHANES: Stage 3 ~ ns(age,2) + age-eligibility + sex + race + reported hypertension + reported diabetes + reported kidney disease + smoking. BRFSS / application: 4,395 training records; 293 cases; 13 coefficients including intercept. No CVD predictor.

No-kidney-report model. NHANES: Primary formula minus reported kidney disease. BRFSS / application: Same training sample; 12 coefficients.

Demographic model. NHANES: Stage 3 ~ ns(age,2) + age-eligibility + sex + race. BRFSS / application: Same training sample; 9 coefficients.

Prediction implementation. NHANES: Preserve training terms and predvars, including age-spline knots, in the model matrix. BRFSS / application: Training-fit reconstruction, split-batch invariance, and explicit matrix-times-coefficient predictions are checked. This rule applies to BRFSS, cross-validation folds, and bootstrap test samples.

BRFSS predictor-completeness IPW uses a weighted quasibinomial response model with age spline (3 degrees of freedom), sex, race/ethnicity and jurisdiction; probabilities are truncated at the complete-case 1st and 99th percentiles. Predictions are normalized within common-CVD-negative respondents. This does not account for excluded respondents whose CVD status is unknown. Table S1 gives the overall flow; Table S4 gives each jurisdiction’s weighted unknown-CVD and predictor-completeness percentages.

Validation uses 20 repetitions of five-fold PSU cross-validation, preserving strata and the prediction basis fitted in each training fold, and 200 stratified PSU bootstrap optimism corrections. Repeated partitions describe internal resampling variability, not external prediction accuracy. Calibration deciles are formed from weighted participant means of out-of-fold predictions; their unweighted counts and cases are shown in Figure S2. Diagnostic observed-proportion bars retain normal-Wald intervals, with boundary groups shown without an interval. Model comparisons use the same training population and frozen age reference.

## M5. State estimands and uncertainty

Within each state’s respondents with known common CVD, let c be reported CVD prevalence and q the normalized IPW mean model prediction among CVD-negative respondents. Reported CVD is c; the modeled contribution is (1−c)q. The two components are shown separately and are not summed as complete advanced-CKM prevalence. This state application does not estimate kidney nonreporting, blood pressure control or glycemic control by state.

Age-standardized components average four age-specific quantities using the fixed pooled 49-jurisdiction BRFSS weights: ages 20–44, 43.4%; 45–64, 32.3%; 65–79, 18.9%; and ≥80, 5.4%. These are displayed to one decimal; calculations use the unrounded weights in Supplementary_Source_Data.xlsx, sheet Additional_outputs, dataset state__age_standardization_reference.csv. The reference is neither the 2000 United States census standard nor an external clinical target population.

For state contribution estimates, first-order delta propagation combines the covariance of BRFSS survey totals with the NHANES coefficient covariance: V=Vs+Vm. The primary conditional interval is estimate ±1.96√V, an asymptotic normal approximation for two independent survey sources. BRFSS sample size alone does not justify neglecting Vm. The weights, fitted response probabilities and age reference are held fixed; structural transport error, estimated response-weight uncertainty, and uncertainty in contextual source estimates are not included.

A targeted finite-degree-of-freedom sensitivity uses ν=(Vs+Vm)²/{Vs²/νs+Vm²/νm}, where νs is each state’s BRFSS design degrees of freedom and νm=15 is the NHANES design degrees of freedom; its critical value is t(0.975,ν). This Welch–Satterthwaite combination is an approximation, not proof of exact coverage for transported estimates. A more conservative stress calculation sets νm=3, the model residual degrees of freedom, to expose the consequence of that choice rather than treating t(3) as automatically correct for the entire two-source estimate.

For standardized stage-3 contributions, model variance contributes 41.1%–97.0% of total variance. The design-degree-of-freedom sensitivity gives effective degrees of freedom 15.9–87.9 and intervals 1.4%–8.2% wider than the normal intervals. Using residual degrees of freedom for the model component widens them by 7.3%–57.1%. Point estimates are unchanged. Figure S2 shows a concrete example; all jurisdictions’ calculations are retained in the Excel source-data workbook. No precision-based ranking or resource-allocation conclusion is drawn.

## M6. Contextual source definitions

Obesity, diabetes, hypertension: Centers for Disease Control and Prevention PLACES 2025 crude county prevalence, aggregated to states with adult population weights; 2025 release. https://data.cdc.gov/resource/swc5-untb

CKD, uninsured: BRFSS 2023 direct state estimates; 2023. https://www.cdc.gov/brfss/annual_data/annual_2023.html

Households below the federal poverty level: America's Health Rankings, sourced from United States Census Bureau American Community Survey one-year estimates; 2024. https://www.americashealthrankings.org/explore/measures/household_poverty

Population in rural areas: America's Health Rankings, sourced from United States Census Bureau American Community Survey one-year estimates; 2023. https://www.americashealthrankings.org/explore/measures/pct_rural_b

Primary care physicians per 100,000 population: Health Resources and Services Administration State of the Primary Care Workforce report; 2025. https://bhw.hrsa.gov/sites/default/files/bureau-health-workforce/data-research/State-of-the-Primary-Care-Workforce-2025.pdf

Household food insecurity: United States Department of Agriculture Economic Research Service Household Food Security in the United States in 2023 (ERR-337); 2021-2023. https://www.ers.usda.gov/publications/109895

Population-weighted county RPL_THEMES: Centers for Disease Control and Prevention/Agency for Toxic Substances and Disease Registry Social Vulnerability Index county layer; 2022. https://onemap.cdc.gov/onemapservices/rest/services/SVI/CDC_ATSDR_Social_Vulnerability_Index_2022_USA/FeatureServer/1

PLACES county estimates are aggregated with county adult population weights (age ≥18); they are model-based source values. Poverty describes households; rurality describes population; food insecurity averages households over 2021–2023. The social vulnerability entry is a population-weighted county percentile score multiplied by 100, not a validated state percentile. Larger physician density indicates more physicians. Some indicators share BRFSS information with the bridge. Mixed periods, denominators, measurement methods and source uncertainty prevent a common score from being interpreted as policy utility. Original components are retained individually in Table S4.

## Supplementary method references

Parker et al. (2017), NCHS Series 2 No.175: https://www.cdc.gov/nchs/data/series/sr_02/sr02_175.pdf. Survey inference: https://cran.r-project.org/web/packages/survey/survey.pdf (svyglm). NHANES design degrees of freedom: https://wwwn.cdc.gov/nchs/nhanes/tutorials/varianceestimation.aspx. Welch–Satterthwaite uncertainty combination: https://www.itl.nist.gov/div898/handbook/mpc/section5/mpc571.htm. PREVENT input conventions: https://martingmayer.com/preventr/reference/estimate_risk. Accessed 7 September 2026. Clinical and data-source references also appear in the main manuscript.

Table S1. Sample selection and indicator response among adults in the United States, National Health and Nutrition Examination Survey, August 2021–August 2023, and Behavioral Risk Factor Surveillance System, 2023

## A. National and state-analysis populations

| Population / step | N | Events / weighted information |
| --- | --- | --- |
| National examination survey: adults with phlebotomy weights and design variables | 5,792 | Base analytic-selection population |
| Excluded for incomplete primary cardiovascular-kidney-metabolic variables | 786 |  |
| Primary national complete cases | 5,006 | 87.1% of weighted eligible population |
| Stage 4 excluded from bridge training | 601 | All stage-4 participants |
| Remaining before additional exclusions | 4,405 | 294 stage-3 cases |
| Unresolved stage (all also lack smoking) | 4 | Excluded from training, retained in national denominator |
| Further missing predictors | 6 | Kidney report: 4 (one stage-3 case); smoking: 2 |
| Final state-model training population | 4,395 | 293 stage-3 cases |
| State telephone survey: adults in 49 jurisdictions with valid positive weights | 418,390 |  |
| Unknown common cardiovascular disease excluded | 4,718 | 1.1% weighted; state range 0.5%–1.8% |
| Known common cardiovascular disease application population | 413,672 | Conditional state denominator |
| Common cardiovascular disease positive | 47,610 | Directly reported component |
| Common cardiovascular disease negative | 366,062 | Predictor-completeness target |
| Predictor-complete cardiovascular disease-negative population | 343,715 | 92.8% of weighted cardiovascular disease-negative population |
| Missing one or more model predictors | 22,347 | Separate from unknown-cardiovascular disease exclusion |

Counts are unweighted and steps are sequential within each survey. NHANES interview/examination response rates were 34.6%/25.7%; BRFSS median weighted American Association for Public Opinion Research Response Rate 4 was 44.6% (range, 31.0%–63.1%). Analytic completeness, unknown outcome responses and survey nonparticipation are distinct selection mechanisms.

## B. Advanced cardiovascular-kidney-metabolic indicators: eligible versus observed denominator

| Indicator | Eligible N | Observed N | Unknown / missing |
| --- | --- | --- | --- |
| Kidney abnormality without reported weak/failing kidneys | 445 | 443 | 2 |
| Blood pressure ≥130/80 mm Hg | 666 | 666 | 0 |
| Glycated hemoglobin ≥7% | 332 | 332 | 0 |
| No reported lipid-lowering medication | 621 | 621 | 0 |
| Low/very low food security among household adults | 895 | 834 | 61 |
| No usual place for care | 895 | 895 | 0 |
| No health insurance | 895 | 895 | 0 |

All events and weighted estimates use the observed denominator. The fixed hypertension-plus-diabetes joint subgroup has 277 observed participants; it is not an all-adults denominator.

Abbreviations: NHANES, National Health and Nutrition Examination Survey; BRFSS, Behavioral Risk Factor Surveillance System; CKM, cardiovascular-kidney-metabolic; CVD, cardiovascular disease; HbA1c, glycated hemoglobin. The national population is aged ≥20 years; the state application uses the same age restriction. Response Rate 4 follows the American Association for Public Opinion Research definition.

Table S2. Definition, threshold and selection sensitivities among adults aged ≥20 years in the United States, National Health and Nutrition Examination Survey, August 2021–August 2023

## A. Weighted stage proportions under specified definitions

| Stage | Primary | Selection inverse-probability weighting | Optional risk inputs omitted | Fasting: primary | Fasting: augmented |
| --- | --- | --- | --- | --- | --- |
| 0 | 13.1 (11.1, 15.4) | 12.9 (10.9, 15.2) | 13.1 (11.1, 15.4) | 15.0 (12.0, 18.5) | 11.4 (9.1, 14.1) |
| 1 | 28.1 (25.8, 30.5) | 27.5 (25.2, 29.8) | 28.1 (25.8, 30.5) | 27.3 (24.9, 29.8) | 24.1 (22.1, 26.1) |
| 2 | 45.8 (43.8, 47.8) | 45.7 (43.7, 47.7) | 45.8 (43.9, 47.7) | 45.4 (43.0, 47.8) | 52.1 (49.4, 54.8) |
| 3 | 4.0 (3.4, 4.8) | 4.3 (3.6, 5.1) | 4.0 (3.3, 4.9) | 3.7 (3.0, 4.5) | 3.8 (3.1, 4.6) |
| 4 | 8.9 (7.8, 10.1) | 9.6 (8.5, 10.9) | 8.9 (7.8, 10.1) | 8.6 (7.3, 10.0) | 8.6 (7.3, 10.0) |
| Unresolved | <0.1 (<0.1, 0.1) | <0.1 (<0.1, 0.1) | <0.1 (<0.1, 0.1) | <0.1 (<0.1, 0.2) | <0.1 (<0.1, 0.2) |
| 3-4 | 13.0 (11.6, 14.4) | 13.9 (12.4, 15.5) | 13.0 (11.6, 14.4) | 12.3 (10.9, 13.7) | 12.3 (10.9, 13.8) |

Cells are percentage (95% CI). The first three columns use N=5,006 and phlebotomy weights (selection-IPW adjusted where shown). The last two use the same N=2,592 and WTSAF2YR; comparisons should be made within that pair. Advanced CKM changes from 12.3% to 12.3% (before rounding, a difference of <0.1 percentage points), while stage 2 changes from 45.4% to 52.1%; this is not evidence that all stages are unchanged. These sensitivities do not replace the primary definition.

## B. Clinical and fixed-subgroup joint indicators by exact stage

| Indicator | Stages 3–4 | Stage 3 | Stage 4 |
| --- | --- | --- | --- |
| Kidney abnormality without reported weak/failing kidneys | 358/443; 80.7 (75.7, 85.1) | 147/184; 79.3 (71.2, 86.0) | 211/259; 81.7 (74.8, 87.3) |
| Blood pressure ≥130/80 mm Hg | 385/666; 58.7 (54.9, 62.5) | 148/225; 70.5 (60.7, 79.0) | 237/441; 53.0 (48.3, 57.8) |
| Glycated hemoglobin ≥7% | 185/332; 56.0 (47.6, 64.1) | 83/129; 65.3 (51.3, 77.6) | 102/203; 50.1 (42.4, 57.7) |
| Blood pressure ≥140/90 mm Hg | 241/666; 36.1 (30.1, 42.4) | 98/225; 46.6 (37.7, 55.7) | 143/441; 31.0 (24.7, 37.9) |
| Glycated hemoglobin ≥8% | 103/332; 31.4 (24.8, 38.6) | 52/129; 41.5 (30.9, 52.7) | 51/203; 25.0 (17.8, 33.4) |
| Both blood pressure ≥130/80 and glycated hemoglobin ≥7%; fixed dual-condition subgroup | 91/277; 34.1 (24.7, 44.4) | 42/107; 43.8 (28.7, 59.8)† | 49/170; 28.0 (20.0, 37.3) |
| Both blood pressure ≥140/90 and glycated hemoglobin ≥8%; fixed dual-condition subgroup | 32/277; 12.6 (7.3, 19.9) | 17/107; 18.1 (8.9, 31.2) | 15/170; 9.2 (5.0, 15.2) |

Each cell gives events/observed N; percentage (95% CI). Blood pressure ≥130/80 or ≥140/90 means systolic OR diastolic elevation; joint rows require both the blood pressure criterion AND the HbA1c criterion in the fixed dual-condition subgroup. A dagger flags limited numerical reliability, especially small stage-3 joint groups. Primary versus higher thresholds use the same condition-defined denominator.

## C. Pooled indicators under selection weighting and optional-input omission

| Indicator | Primary | Selection inverse-probability weighting | Optional biomarker inputs omitted |
| --- | --- | --- | --- |
| Kidney abnormality without reported weak/failing kidneys | 358/443; 80.7 (75.7, 85.1) | 358/443; 80.1 (74.9, 84.6) | 336/422; 79.4 (74.1, 84.1) |
| Blood pressure ≥130/80 mm Hg | 385/666; 58.7 (54.9, 62.5) | 385/666; 59.0 (55.1, 62.8) | 395/679; 58.8 (55.0, 62.6) |
| Glycated hemoglobin ≥7% | 185/332; 56.0 (47.6, 64.1) | 185/332; 55.6 (47.5, 63.6) | 166/325; 51.0 (42.8, 59.2) |
| No reported lipid-lowering medication | 115/621; 18.6 (14.7, 23.0) | 115/621; 18.4 (14.3, 23.2) | — |
| Low/very low food security among household adults | 164/834; 18.8 (14.8, 23.2) | 164/834; 19.4 (15.4, 24.0) | — |
| No usual place for care | 33/895; 4.0 (2.3, 6.2) | 33/895; 3.9 (2.3, 6.1) | — |
| No health insurance | 27/895; 3.3 (2.0, 5.1) | 27/895; 3.5 (2.1, 5.4) | — |
| Both blood pressure ≥130/80 and glycated hemoglobin ≥7%; fixed dual-condition subgroup | 91/277; 34.1 (24.7, 44.4) | 91/277; 33.6 (24.5, 43.7) | 86/275; 31.8 (23.2, 41.5) |
| Both blood pressure ≥140/90 and glycated hemoglobin ≥8%; fixed dual-condition subgroup | 32/277; 12.6 (7.3, 19.9) | 32/277; 12.4 (7.2, 19.3) | 28/275; 11.0 (5.7, 18.6) |

Optional-input omission holds primary clinical thresholds, observed-response rules and age/boundary conventions fixed but reclassifies stages. It is a specified sensitivity for the clinical markers, not an alternative primary analysis. Stage-3 HbA1c ≥7% decreases from 65.3% to 52.6%, with stage 4 unchanged at 50.1%; pooled blood pressure is 58.8% and joint elevation 31.8%. This sensitivity illustrates definition dependence and does not remove it. Contextual endpoints were not recalculated under optional-input omission.

Kidney-definition sensitivity: Supplementary_Source_Data.xlsx, sheet Kidney_sensitivity, reports all seven single-visit kidney-marker definitions under complete-case and inverse-probability weighting, including the more severe definitions. These alternative abnormality definitions do not establish chronic kidney disease. The main kidney result remains KIQ022 nonreporting among the prespecified single-visit abnormality denominator.

Abbreviations: CI, confidence interval; CKM, cardiovascular-kidney-metabolic; HbA1c, glycated hemoglobin; IPW, inverse-probability weighting; PREVENT, Predicting Risk of Cardiovascular Disease Events; UACR, urine albumin-to-creatinine ratio. WTSAF2YR is the survey fasting-subsample weight. All intervals use comma-separated lower and upper limits; “<0.1” preserves small positive values rather than displaying zero.

Table S3. Socioeconomic associations and support for stage comparisons among adults aged ≥20 years in the United States, National Health and Nutrition Examination Survey, August 2021–August 2023

## A. Socioeconomic prevalence ratios on the same complete-case population

| Comparison | Demographics + one social factor: prevalence ratio (95% confidence interval) | Full model: prevalence ratio (95% confidence interval) |
| --- | --- | --- |
| College graduate (reference) | 1.00 | 1.00 |
| Some college/associate degree vs college graduate+ | 1.49 (1.23, 1.82) | 1.31 (1.07, 1.60) |
| High school/equivalent vs college graduate+ | 1.75 (1.40, 2.19) | 1.47 (1.20, 1.80) |
| <High school vs college graduate+ | 2.23 (1.80, 2.77) | 1.84 (1.42, 2.37) |
| Poverty income ratio ≥3.50 (reference) | 1.00 | 1.00 |
| poverty income ratio 1.30–3.49 vs ≥3.50 | 1.40 (1.22, 1.60) | 1.36 (1.16, 1.59) |
| poverty income ratio <1.30 vs ≥3.50 | 2.04 (1.64, 2.54) | 1.63 (1.24, 2.13) |
| Health insurance (reference) | 1.00 | 1.00 |
| No health insurance vs insured | 0.73 (0.43, 1.23) | 0.48 (0.31, 0.75) |
| Full/marginal adult food security (reference) | 1.00 | 1.00 |
| Low/very low adult food security vs full/marginal | 1.77 (1.39, 2.25) | 1.21 (0.91, 1.63) |

N=4,382; advanced-CKM cases=770. The full model adjusts for all listed social factors plus age group, sex and race/ethnicity; demographic models adjust for an age spline (2 degrees of freedom), sex and race/ethnicity and one factor. Both use individual-coefficient t(15) intervals. They are descriptive and are not selected by significance. See M3 for differing age specifications, evidence hierarchy and inferential limitations.

## B. Equity model size and design information

| Model | N / events | Slopes | Design degrees of freedom | Residual degrees of freedom | Covariance rank |
| --- | --- | --- | --- | --- | --- |
| Full | 4382/770 | 16 | 15 | -1 | 15 |
| Demographics + education | 4382/770 | 11 | 15 | 4 | 12 |
| Demographics + poverty income ratio | 4382/770 | 10 | 15 | 5 | 11 |
| Demographics + insurance | 4382/770 | 9 | 15 | 6 | 10 |
| Demographics + food security | 4382/770 | 9 | 15 | 6 | 10 |

All five models converged. A negative residual degrees of freedom and rank-deficient full covariance preclude a full-parameter joint test; the displayed individual intervals use the stated design-degree-of-freedom approximation, not negative-degrees of freedom t intervals. Complete coefficient outputs remain in the Excel source-data workbook.

## C. Support restriction for adjusted stage comparisons

| Applicable indicator | Stage 3 | Stage 4 |
| --- | --- | --- |
| Kidney abnormality without reported weak/failing kidneys | 179/184; 5 excluded (2.2% weighted) | 200/259; 59 excluded (26.8% weighted) |
| Blood pressure ≥130/80 mm Hg | 221/225; 4 excluded (1.5% weighted) | 370/441; 71 excluded (18.3% weighted) |
| Glycated hemoglobin ≥7% | 129/129; 0 excluded (0.0% weighted) | 173/203; 30 excluded (15.2% weighted) |
| Both blood pressure ≥130/80 and glycated hemoglobin ≥7%; fixed dual-condition subgroup | 107/107; 0 excluded (0.0% weighted) | 142/170; 28 excluded (17.0% weighted) |

Restriction: ages 45–79 and sex-by-race/ethnicity cells observed in both stages; percentages are excluded weighted shares. Higher thresholds use the same condition-defined samples. Pooled/restricted models have nine/eight slopes and six/seven residual degrees of freedom, respectively (Figure S1). The higher-threshold pooled joint fit is unreliable and not interpreted. IPW contrasts remain exploratory; interval inclusion of zero does not establish robustness.

Abbreviations: CI, confidence interval; CKM, cardiovascular-kidney-metabolic; PR, prevalence ratio; PIR, poverty income ratio. Reference categories are 1.00. Counts are unweighted; estimates are survey weighted.

Table S4. Disease components among adults and contextual profiles for 48 states and the District of Columbia, United States, 2021–2025

## A. Age-standardized state components, conditional uncertainty and missingness

| Jurisdiction | Reported cardiovascular disease, % (95% confidence interval) | Modeled stage-3 contribution, % (95% confidence interval) | Unknown disease status, % | Predictor complete, % | Stage-3 standard error, points Survey / model |
| --- | --- | --- | --- | --- | --- |
| Alabama | 12.5 (11.4, 13.6) | 5.9 (4.8, 6.9) | 0.8 | 96.3 | 0.2 / 0.5 |
| Alaska | 9.0 (8.0, 10.0) | 4.6 (3.9, 5.3) | 1.5 | 94.1 | 0.2 / 0.3 |
| Arizona | 8.2 (7.5, 8.8) | 5.4 (4.7, 6.1) | 1.0 | 94.6 | 0.2 / 0.3 |
| Arkansas | 12.3 (11.3, 13.3) | 5.3 (4.5, 6.1) | 1.7 | 93.4 | 0.2 / 0.4 |
| California | 7.8 (7.1, 8.5) | 5.5 (4.7, 6.3) | 1.2 | 91.4 | 0.2 / 0.4 |
| Colorado | 7.3 (6.6, 8.0) | 4.5 (3.9, 5.1) | 0.8 | 93.7 | 0.2 / 0.3 |
| Connecticut | 7.6 (6.8, 8.5) | 5.0 (4.3, 5.7) | 1.3 | 91.6 | 0.2 / 0.3 |
| Delaware | 8.9 (7.8, 9.9) | 5.2 (4.3, 6.2) | 0.9 | 92.5 | 0.2 / 0.4 |
| District of Columbia | 7.0 (5.8, 8.3) | 6.5 (4.9, 8.1) | 1.2 | 92.7 | 0.3 / 0.7 |
| Florida | 10.1 (9.0, 11.1) | 5.3 (4.5, 6.0) | 1.1 | 92.2 | 0.2 / 0.3 |
| Georgia | 9.4 (8.6, 10.2) | 6.1 (5.0, 7.2) | 1.1 | 90.5 | 0.2 / 0.5 |
| Hawaii | 7.8 (6.9, 8.7) | 6.0 (3.7, 8.3) | 0.8 | 94.9 | 0.2 / 1.2 |
| Idaho | 7.9 (7.1, 8.6) | 4.7 (4.0, 5.4) | 1.0 | 95.9 | 0.2 / 0.3 |
| Illinois | 8.2 (7.2, 9.2) | 5.5 (4.6, 6.3) | 1.2 | 94.4 | 0.3 / 0.4 |
| Indiana | 10.5 (9.9, 11.2) | 5.5 (4.7, 6.3) | 1.1 | 90.6 | 0.1 / 0.4 |
| Iowa | 8.2 (7.6, 8.9) | 5.2 (4.4, 6.0) | 0.8 | 95.6 | 0.2 / 0.4 |
| Kansas | 8.9 (8.2, 9.6) | 5.0 (4.3, 5.7) | 1.3 | 94.3 | 0.2 / 0.3 |
| Louisiana | 12.2 (11.1, 13.3) | 6.1 (4.9, 7.2) | 1.1 | 91.8 | 0.2 / 0.5 |
| Maine | 9.6 (8.9, 10.2) | 4.7 (4.0, 5.4) | 0.9 | 93.9 | 0.1 / 0.3 |
| Maryland | 8.2 (7.6, 8.9) | 5.6 (4.6, 6.5) | 1.0 | 92.5 | 0.1 / 0.5 |
| Massachusetts | 7.6 (6.9, 8.4) | 5.0 (4.3, 5.8) | 0.9 | 91.6 | 0.2 / 0.3 |
| Michigan | 9.8 (9.1, 10.5) | 5.5 (4.7, 6.3) | 1.1 | 93.3 | 0.2 / 0.4 |
| Minnesota | 8.5 (7.8, 9.1) | 4.7 (4.0, 5.4) | 1.2 | 92.0 | 0.1 / 0.3 |
| Mississippi | 11.6 (10.3, 12.9) | 6.4 (5.0, 7.7) | 1.6 | 95.5 | 0.3 / 0.6 |
| Missouri | 10.9 (10.0, 11.8) | 5.2 (4.4, 6.0) | 1.2 | 95.1 | 0.2 / 0.4 |
| Montana | 8.3 (7.6, 9.0) | 4.4 (3.8, 5.1) | 1.0 | 96.3 | 0.1 / 0.3 |
| Nebraska | 8.0 (7.4, 8.5) | 5.1 (4.4, 5.9) | 0.7 | 94.5 | 0.1 / 0.3 |
| Nevada | 8.3 (6.0, 10.5) | 5.8 (4.8, 6.8) | 1.4 | 93.2 | 0.4 / 0.3 |
| New Hampshire | 7.2 (6.5, 7.8) | 4.7 (4.0, 5.4) | 0.9 | 94.8 | 0.1 / 0.3 |
| New Jersey | 6.8 (6.2, 7.4) | 5.2 (4.4, 5.9) | 0.9 | 91.9 | 0.2 / 0.3 |
| New Mexico | 8.5 (7.0, 10.1) | 5.3 (4.5, 6.1) | 0.7 | 94.9 | 0.3 / 0.3 |
| New York | 8.3 (7.6, 8.9) | 5.1 (4.4, 5.8) | 1.1 | 91.4 | 0.2 / 0.3 |
| North Carolina | 10.2 (8.9, 11.5) | 5.6 (4.6, 6.6) | 0.5 | 95.6 | 0.3 / 0.4 |
| North Dakota | 8.7 (7.8, 9.5) | 4.8 (4.0, 5.5) | 0.9 | 92.5 | 0.2 / 0.3 |
| Ohio | 9.8 (9.2, 10.4) | 5.4 (4.6, 6.2) | 1.0 | 93.6 | 0.1 / 0.4 |
| Oklahoma | 10.9 (10.0, 11.7) | 5.0 (4.3, 5.8) | 1.8 | 92.8 | 0.2 / 0.3 |
| Oregon | 8.4 (7.6, 9.3) | 4.8 (4.1, 5.5) | 1.3 | 93.7 | 0.2 / 0.3 |
| Rhode Island | 8.9 (7.9, 9.8) | 5.1 (4.3, 5.9) | 1.0 | 93.4 | 0.2 / 0.3 |
| South Carolina | 10.3 (9.5, 11.0) | 5.7 (4.7, 6.7) | 1.1 | 92.3 | 0.1 / 0.5 |
| South Dakota | 8.9 (7.4, 10.5) | 4.8 (3.8, 5.7) | 0.7 | 92.9 | 0.3 / 0.3 |
| Tennessee | 12.3 (11.1, 13.4) | 5.5 (4.6, 6.5) | 1.6 | 92.8 | 0.2 / 0.4 |
| Texas | 9.0 (8.1, 9.9) | 5.5 (4.7, 6.2) | 0.9 | 92.6 | 0.2 / 0.3 |
| Utah | 7.5 (6.9, 8.2) | 4.5 (3.8, 5.1) | 1.3 | 94.3 | 0.1 / 0.3 |
| Vermont | 7.4 (6.6, 8.1) | 4.4 (3.6, 5.1) | 1.0 | 93.7 | 0.2 / 0.3 |
| Virginia | 10.0 (9.2, 10.9) | 5.3 (4.4, 6.1) | 0.8 | 93.1 | 0.2 / 0.4 |
| Washington | 7.5 (7.1, 7.9) | 4.8 (4.2, 5.4) | 0.9 | 95.6 | 0.1 / 0.3 |
| West Virginia | 13.5 (12.4, 14.6) | 5.3 (4.4, 6.2) | 1.6 | 96.2 | 0.2 / 0.4 |
| Wisconsin | 9.2 (8.7, 9.8) | 4.9 (4.1, 5.6) | 1.0 | 90.5 | 0.1 / 0.3 |
| Wyoming | 8.8 (8.0, 9.7) | 4.4 (3.8, 5.1) | 0.9 | 94.9 | 0.1 / 0.3 |

CVD and modeled stage-3 estimates use known common-CVD respondents. Unknown-CVD percentage uses all otherwise eligible BRFSS respondents in that jurisdiction; predictor-complete percentage uses common-CVD-negative respondents. SEs are percentage points and propagate independent BRFSS survey and NHANES coefficient variance. Intervals use the normal critical value 1.96; finite-degree-of-freedom sensitivities appear in M5 and Figure S2. The fixed age reference is given in M5. Components are not added, and states are ordered alphabetically. Crude estimates, response diagnostics and all interval-sensitivity calculations are supplied as Excel source-data workbook.

## B. Cardiometabolic and insurance descriptors in original percentages

| Jurisdiction | Obesity | Diabetes | Hypertension | Reported kidney disease | Uninsured |
| --- | --- | --- | --- | --- | --- |
| Alabama | 39.4 | 14.8 | 43.3 | 4.8 | 7.6 |
| Alaska | 35.5 | 9.4 | 32.5 | 2.6 | 6.8 |
| Arizona | 31.3 | 11.3 | 32.8 | 4.9 | 8.6 |
| Arkansas | 39.3 | 13.3 | 41.4 | 4.5 | 8.8 |
| California | 27.8 | 11.7 | 30.5 | 3.5 | 5.4 |
| Colorado | 26.1 | 8.6 | 27.0 | 3.4 | 7.4 |
| Connecticut | 30.5 | 10.2 | 32.0 | 3.5 | 5.5 |
| Delaware | 35.3 | 13.1 | 38.1 | 5.0 | 4.5 |
| District of Columbia | 24.7 | 7.8 | 28.5 | 2.4 | 2.8 |
| Florida | 31.9 | 13.5 | 36.6 | 5.2 | 9.2 |
| Georgia | 35.2 | 13.3 | 37.4 | 4.0 | 11.0 |
| Hawaii | 25.6 | 12.4 | 31.5 | 4.3 | 2.3 |
| Idaho | 32.1 | 10.1 | 31.5 | 4.2 | 7.1 |
| Illinois | 34.0 | 11.7 | 33.0 | 3.8 | 5.6 |
| Indiana | 38.4 | 13.0 | 37.8 | 5.0 | 6.1 |
| Iowa | 37.7 | 11.2 | 33.9 | 4.1 | 4.7 |
| Kansas | 36.5 | 11.4 | 34.4 | 3.6 | 6.8 |
| Louisiana | 40.0 | 15.2 | 42.6 | 4.5 | 4.7 |
| Maine | 32.7 | 11.2 | 35.7 | 4.2 | 5.5 |
| Maryland | 35.1 | 12.5 | 36.8 | 3.7 | 5.1 |
| Massachusetts | 27.7 | 9.8 | 30.9 | 3.5 | 2.2 |
| Michigan | 36.2 | 11.9 | 36.7 | 4.4 | 3.8 |
| Minnesota | 33.5 | 10.1 | 31.0 | 3.7 | 4.5 |
| Mississippi | 40.4 | 15.5 | 44.9 | 4.1 | 9.2 |
| Missouri | 36.6 | 12.1 | 36.5 | 4.3 | 5.6 |
| Montana | 31.4 | 9.9 | 31.9 | 3.4 | 7.1 |
| Nebraska | 37.7 | 10.7 | 32.6 | 3.2 | 6.6 |
| Nevada | 31.3 | 12.0 | 34.0 | 7.5 | 12.0 |
| New Hampshire | 31.8 | 9.4 | 33.0 | 3.2 | 4.1 |
| New Jersey | 28.9 | 10.9 | 32.8 | 3.6 | 7.7 |
| New Mexico | 34.1 | 13.2 | 34.3 | 4.0 | 9.6 |
| New York | 28.8 | 11.5 | 32.6 | 3.1 | 4.4 |
| North Carolina | 35.1 | 12.0 | 36.8 | 4.1 | 7.1 |
| North Dakota | 36.5 | 9.8 | 31.8 | 4.1 | 4.2 |
| Ohio | 37.1 | 13.2 | 37.2 | 5.0 | 5.4 |
| Oklahoma | 38.9 | 12.8 | 38.5 | 4.5 | 8.3 |
| Oregon | 33.6 | 11.2 | 32.7 | 4.1 | 5.3 |
| Rhode Island | 31.7 | 11.1 | 33.8 | 3.8 | 4.2 |
| South Carolina | 35.8 | 13.9 | 38.5 | 4.9 | 8.4 |
| South Dakota | 35.9 | 11.7 | 33.3 | 3.7 | 6.6 |
| Tennessee | 37.6 | 13.6 | 39.5 | 5.4 | 8.2 |
| Texas | 35.6 | 12.7 | 33.1 | 3.7 | 16.0 |
| Utah | 31.0 | 8.5 | 27.3 | 3.4 | 7.3 |
| Vermont | 28.6 | 8.8 | 31.4 | 3.2 | 3.5 |
| Virginia | 34.5 | 12.5 | 35.4 | 4.7 | 6.7 |
| Washington | 30.6 | 9.5 | 30.5 | 3.7 | 5.9 |
| West Virginia | 40.6 | 15.9 | 44.0 | 5.5 | 5.1 |
| Wisconsin | 37.7 | 11.4 | 33.3 | 4.1 | 5.3 |
| Wyoming | 33.7 | 10.9 | 31.7 | 3.4 | 10.1 |

## C. Social and access descriptors in original source units

| Jurisdiction | Household poverty, % | Rural population, % | Primary-care physicians / 100,000 | Household food insecurity, % | Mean county social vulnerability score, 0–100 |
| --- | --- | --- | --- | --- | --- |
| Alabama | 15.9 | 42.0 | 82.4 | 11.5 | 62.4 |
| Alaska | 8.7 | 35.1 | 117.2 | 10.4 | 57.1 |
| Arizona | 11.6 | 11.5 | 83.2 | 11.8 | 76.7 |
| Arkansas | 16.0 | 43.9 | 88.8 | 18.9 | 68.0 |
| California | 11.9 | 5.8 | 109.7 | 11.4 | 72.3 |
| Colorado | 9.8 | 14.4 | 107.1 | 9.9 | 41.8 |
| Connecticut | 11.0 | 13.8 | 115.7 | 10.4 | 54.8 |
| Delaware | 10.0 | 17.9 | 106.0 | 11.3 | 57.0 |
| District of Columbia | 15.0 | 0.0 | 249.4 | 8.8 | 62.6 |
| Florida | 12.3 | 9.1 | 94.4 | 12.0 | 72.2 |
| Georgia | 12.9 | 26.6 | 86.6 | 12.8 | 63.6 |
| Hawaii | 10.4 | 14.0 | 122.5 | 9.6 | 56.2 |
| Idaho | 10.9 | 30.8 | 75.2 | 11.3 | 40.9 |
| Illinois | 12.2 | 13.2 | 109.8 | 12.4 | 54.9 |
| Indiana | 12.4 | 28.9 | 82.8 | 12.2 | 45.7 |
| Iowa | 11.3 | 37.3 | 90.4 | 9.8 | 30.3 |
| Kansas | 11.3 | 28.2 | 98.0 | 10.6 | 43.5 |
| Louisiana | 18.8 | 29.1 | 93.4 | 16.2 | 77.6 |
| Maine | 11.6 | 61.5 | 132.2 | 10.9 | 30.0 |
| Maryland | 10.0 | 14.7 | 126.7 | 10.4 | 47.3 |
| Massachusetts | 10.8 | 8.9 | 147.4 | 9.3 | 47.0 |
| Michigan | 13.5 | 27.1 | 107.9 | 13.0 | 44.7 |
| Minnesota | 10.2 | 28.9 | 114.1 | 9.1 | 31.9 |
| Mississippi | 18.2 | 54.4 | 70.0 | 16.2 | 75.3 |
| Missouri | 12.9 | 30.8 | 98.0 | 12.7 | 44.3 |
| Montana | 10.6 | 47.1 | 97.7 | 10.6 | 30.5 |
| Nebraska | 11.3 | 28.1 | 97.1 | 12.9 | 40.2 |
| Nevada | 11.8 | 6.2 | 75.7 | 12.5 | 80.8 |
| New Hampshire | 8.2 | 42.4 | 106.7 | 7.4 | 17.0 |
| New Jersey | 9.7 | 6.3 | 107.0 | 9.8 | 56.4 |
| New Mexico | 16.7 | 24.7 | 97.0 | 12.9 | 81.5 |
| New York | 14.2 | 12.9 | 123.5 | 12.3 | 62.4 |
| North Carolina | 12.8 | 33.3 | 92.1 | 10.9 | 62.7 |
| North Dakota | 11.3 | 39.3 | 98.2 | 8.6 | 27.4 |
| Ohio | 13.0 | 24.0 | 106.7 | 12.8 | 45.7 |
| Oklahoma | 15.3 | 35.8 | 75.6 | 15.4 | 72.0 |
| Oregon | 12.3 | 19.7 | 117.1 | 12.8 | 58.8 |
| Rhode Island | 12.9 | 9.7 | 129.9 | 9.7 | 56.3 |
| South Carolina | 13.8 | 31.8 | 86.1 | 14.4 | 65.3 |
| South Dakota | 10.7 | 42.3 | 102.1 | 9.0 | 35.1 |
| Tennessee | 13.2 | 34.7 | 89.3 | 11.7 | 55.3 |
| Texas | 13.3 | 17.0 | 79.5 | 16.9 | 76.7 |
| Utah | 8.5 | 10.8 | 71.8 | 11.8 | 33.0 |
| Vermont | 9.9 | 66.1 | 146.6 | 9.2 | 22.6 |
| Virginia | 10.1 | 24.9 | 100.5 | 10.0 | 38.7 |
| Washington | 10.0 | 16.6 | 110.1 | 9.5 | 49.1 |
| West Virginia | 17.5 | 55.2 | 107.7 | 13.7 | 45.0 |
| Wisconsin | 10.8 | 33.6 | 101.4 | 10.7 | 29.0 |
| Wyoming | 11.3 | 37.4 | 83.6 | 13.1 | 31.7 |

Panel A includes adults aged ≥20 years in 2023 and a model trained in August 2021–August 2023. Panels B–C retain the source-specific populations and periods detailed in M6; measurement methods and precision differ. These descriptors do not form a policy ranking. CI, confidence interval; CVD, cardiovascular disease; SE, standard error; BRFSS, Behavioral Risk Factor Surveillance System; NHANES, National Health and Nutrition Examination Survey.

Figure S1. Joint clinical indicators and adjusted stage comparisons among adults in the United States, National Health and Nutrition Examination Survey, August 2021–August 2023

Panel A describes the same fixed hypertension-plus-diabetes subgroup under primary and higher thresholds. Events and observed totals are unweighted; proportions and Korn–Graubard intervals are survey weighted. Panel B compares adjusted stage 3-minus-stage 4 differences for the pooled stage 2–4 target and restricted stage 3–4 target, with 6 and 7 residual degrees of freedom, respectively. The restricted target is ages 45–79 in sex-by-race/ethnicity cells observed in both stages. The joint interval in that target includes zero. These targets differ, and their contrast is not a formal interaction test. Both comparisons adjust for age, sex and race/ethnicity using complete-case weights. HbA1c denotes glycated hemoglobin; a dagger flags limited numerical reliability. Table S3 gives sample exclusions.

Figure S2. Internal model performance among United States adults, National Health and Nutrition Examination Survey, August 2021–August 2023, and interval sensitivity in the Behavioral Risk Factor Surveillance System, 2023

Calibration groups are weighted deciles of repeated out-of-fold predictions. Cases and totals are unweighted; predicted and observed values are weighted percentages. Diagnostic bars use normal-Wald intervals; boundary groups have no interval. Models use the same 4,395 participants (293 cases) and fixed age reference. Validation used 20 repetitions of five-fold cross-validation and 200 bootstraps. Area under the receiver operating characteristic curve, Brier score and calibration slope are scaled by 100; bootstrap-corrected area is 96.2. Maximum state differences are percentage points. Panel D uses one District of Columbia estimate; M5 details the approximations and excluded uncertainties. Internal diagnostics do not establish external validity against measured state cardiovascular-kidney-metabolic syndrome.
